# 

**Published:** 1849-10

**Authors:** 


### WESTERN JOURNAL

OF

### MEDICINE AND SURGERY:

EDITED BY

**LUNSFORD P. YANDELL, M. D.,**

PROFESSOR OF PHYSIOLOGY AND PATHOLOGICAL ANATOMY IN THE UNIVERSITY OF LOUISVILLE,

AND

**THEODORE S. BELL, M. D.**

---

CXVIII.—OCTOBER, 1849.

---

**THIRD SERIES.**

VOL. IV.....NO. IV.

---

LOUISVILLE, KY:

PUBLISHED MONTHLY BY PRENTICE AND WEISSINGER,

PROPRIETORS AND PRINTERS.

1849.

[POSTAGE 6¼ CENTS.]

